# The Intersection of Offline Learning and Rehabilitation

**DOI:** 10.3389/fnhum.2021.667574

**Published:** 2021-04-21

**Authors:** Brian P. Johnson, Leonardo G. Cohen, Kelly P. Westlake

**Affiliations:** ^1^Department of Physical Therapy and Rehabilitation Science, School of Medicine, University of Maryland, Baltimore, MD, United States; ^2^Human Cortical Physiology and Neurorehabilitation Section, National Institute of Neurological Disorders and Stroke, Bethesda, MD, United States

**Keywords:** occupational therapy, physical therapy, neurorehabilitation, motor learning, memory consolidation

## Introduction

Learning is fundamental to rehabilitation (Krakauer, 2006). The learning of cognitive and motor tasks similar to those in rehabilitative services (i.e., occupational therapy, physical therapy, speech language pathology, recreational therapy, music therapy, etc.) involve the creation (or modification) of neural representations associated with task performance (Dayan and Cohen, 2011). Later accessing these representations allows for performance with greater skill. These neural representations can therefore be referred to as memory traces, and rehabilitation can be thought of as involving the creation and/or modification of memories that can be stored for use in other contexts in the future.

The process of learning is comprised of practice-dependent (i.e., online) and practice-independent (i.e., offline) processes. Skill acquisition during initial practice is typically exhibited by fast improvements in performance (Dayan and Cohen, 2011). After encoding a memory and halting practice, a memory can then undergo consolidation, leading to slower improvements over a period of seconds, days, weeks, or months. The purpose of this paper is two-fold: to identify the currently known mechanisms of consolidation and reconsolidation that impact learning, and to discuss how these findings could impact the design and optimization of interventions and strategies for rehabilitation services. The concepts discussed in this paper are applicable to various forms of learning (e.g., cognitive, motor, visual perceptual) but for simplicity, many of the studies highlighted in this paper involve motor learning.

### Consolidation

Consolidation involves the stabilization (Brashers-Krug et al., 1996; Yotsumoto et al., 2009; Censor et al., 2010; Cohen and Robertson, 2011) or enhancement (Karni et al., 1994; Stickgold et al., 2000; Walker et al., 2002; Fischer et al., 2005; Korman et al., 2007; Nishida and Walker, 2007) of performance across a period of wakeful rest or sleep. The time period for consolidation to occur is typically over hours or perhaps longer based on the complexity of the task, which is referred to here as slow consolidation. More recently though, evidence of rapid within-session consolidation has been identified during the seconds of rest between trials of motor practice (Bönstrup et al., 2019, 2020).

Both implicit and explicit learning involve consolidation. While time alone (i.e., regardless of being awake or asleep) is sufficient for implicit aspects of memory, a period of sleep is necessary for slow consolidation of explicit aspects of memory (Robertson et al., 2004; Albouy et al., 2013, 2015).

The degree of consolidation over a sleep period has been associated with the number of occurrences of sleep spindles and slow wave electroencephalographic waveforms, which predominantly occur during non-rapid eye movement sleep over task-related brain regions (Nishida and Walker, 2007; Barakat et al., 2013; Tamaki et al., 2013). However, the number of sleep spindles and slow waves experienced during sleep decreases with age, which may explain the decrease in sleep-based consolidation found in older adults (Brown et al., 2009; Wilson et al., 2012; Fogel et al., 2014; Roig et al., 2014) and in individuals with sleep apnea (Djonlagic et al., 2012, 2015; Landry et al., 2014; Johnson et al., 2019b).

### Reconsolidation

When later recalling (or performing) a memory that has been consolidated through slow consolidation, online and offline processes can occur again to further fine tune recall (or performance) of the memory, known as reconsolidation (Nader et al., 2000; Walker et al., 2003; Forcato et al., 2007; Lee, 2008; Sandrini et al., 2015; Amar-Halpert et al., 2017; Herszage and Censor, 2018) but see Hardwicke et al. (2016). Gradual session-by-session improvements of a previously acquired and consolidated task may be promoted by reconsolidation between sessions, which is triggered by practice-induced memory reactivation during the session (Censor et al., 2010) or even the presentation of a task-associated sensory cue without active practice (Bavassi et al., 2019). The process of fine-tuning a memory through reconsolidation necessitates the integration of new task information obtained during reactivation so that memories can remain relevant and effective. Such new information may be in the form of sensorimotor calibrations, contextual cues, or additional declarative information. Less is known about rapid reconsolidation during early skill learning (Bönstrup et al., 2019, 2020).

### Interference of Consolidation and Reconsolidation

**Figure 1:**
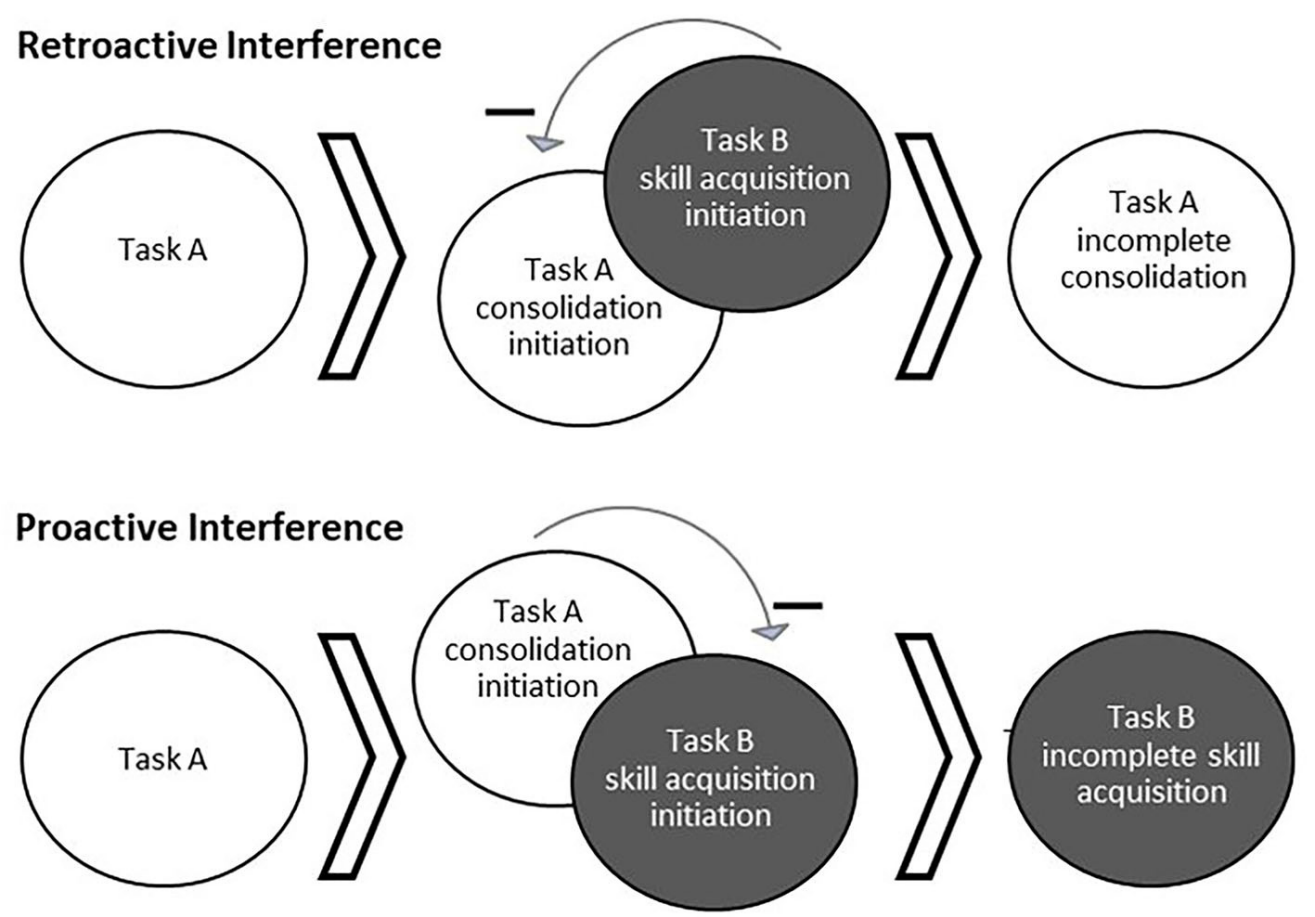
Behaviorally-induced retroactive and proactive interference. Interference occurs when the processes for learning multiple tasks interact and cause a detriment to the consolidation or acquisition of one of the tasks. Top: Acquisition of a second task (Task B) while a first task (Task A) is still undergoing consolidation can result in interference of Task A consolidation, known as retroactive interference. Bottom: Alternatively, ongoing consolidation of a first task (Task A) can interfere with the acquisition of a second task (Task B), known as proactive interference.

Memories are unstable while undergoing consolidation and reconsolidation and are thus susceptible to interference (Figure 1), making subsequent behavior and sleep between sessions crucial to learning (Walker et al., 2003; Forcato et al., 2007; Lee, 2008; Censor et al., 2010). When motor task A is acquired and the consolidation process has begun, the subsequent learning of a different task, task B, can impair consolidation of task A such that later recall performance of task A is impaired. This is known as retroactive interference (Shadmehr and Brashers-Krug, 1997; Ghilardi et al., 2009). For example, learning two different motor tasks within 5 min, 30 min, or 2.5 h was found to induce forgetting of the first motor task learned relative to a gap of 5.5 or 24 h (Shadmehr and Brashers-Krug, 1997). Alternatively, proactive interference can occur when the consolidation process of an initial task can temporarily impair learning of a different task (Ghilardi et al., 2009; Cantarero et al., 2013). For example, Cantarero et al. (2013) found that transiently increased cortical excitability induced through learning an initial motor skill interfered with immediate learning of a second motor skill. However, no retroactive or proactive interference was found if cortical excitability was allowed to return to baseline over time (Cantarero et al., 2013). It should be noted that interference can occur between different task types (i.e., cognitive and motor) (Brown and Robertson, 2007; Mutanen et al., 2020). The topic of memory modification during instability extends to reconsolidation as well. For example, implementing reward during memory reactivation (wherein no reward was present during initial learning) has been found to disrupt reconsolidation, possibly by creating a competing memory trace (Dayan et al., 2016). Whether interference of skill occurs relates to the degree of memory stability when beginning to learn the subsequent task, as well as the similarity of the tasks.

### Preventing Interference Effects

An unstable memory can be modulated (and generalized) more easily, whereas a stable memory is harder to modulate. There are two primary factors that have been found to enable memories to stabilize. The first factor is the amount/duration of practice. Increasing the number of repetitions of task A helps to stabilize a memory trace, thereby reducing retroactive interference. However, increased repetitions of Task A can also transiently increase proactive interference to a subsequently learned task (i.e., Task B) as Task A is being consolidated (Krakauer et al., 2005; Shibata et al., 2017). This retroactive protective effect of increased practice duration extends to reconsolidation as well, as increasing the length of time during which the memory is reactivated decreases retroactive interference (de Beukelaar et al., 2014).

Second, the duration between sessions of learning helps to stabilize memories via consolidation. Allowing for several hours between task practice has been shown to decrease both retroactive and proactive interference compared to a period of several minutes between tasks (Walker et al., 2003; Krakauer et al., 2005; Ghilardi et al., 2009). Including a period of sleep between task practice sessions also reduces the proactive and retroactive interference between two tasks and lessens the amount of time required for consolidation compared to waking hours (Ellenbogen et al., 2006, 2009; Abel and Bäuml, 2014), but see Bailes et al. (2020). For reconsolidation, Gabitov et al. (2017) found that learning of a new motor task caused retroactive interference immediately after memory reactivation, but not if an 8-h interval was afforded following memory reactivation. Others have reported that the role of retroactive interference is greatest immediately after memory reactivation (i.e., 0 s) and fades in magnitude over a short period of time (i.e., 20, 40, and 60 s) as the memory is being reconsolidated (de Beukelaar et al., 2016).

## Discussion

### Consolidation and Reconsolidation During Rehabilitation

While consolidation and reconsolidation are relevant to psychotherapy treatments such as the extinction of fear memories (Monfils et al., 2009; Schiller et al., 2010), it remains to be seen whether rehabilitation services trigger consolidation and reconsolidation. There are long held principles that may make the consolidation and reconsolidation of memories during and after rehabilitation likely. For example, the notion of the “just right challenge,” holds that tasks performed during rehabilitation should be meaningful and difficult (Ayres, 1983; Csikszentmihalyi and LeFevre, 1989; Moneta and Csikszentmihalyi, 1996; Csikszentmihalyi, 2000), and should incorporate learning principles (e.g., practice structure, repetition, feedback, reward) (Poole, 1991; Jarus, 1994). Motor learning concepts such as goal-oriented training or task-specific training are important for skill acquisition during sessions, but interference between-sessions may occur and requires further investigation. That is, individuals receiving goal-oriented training or task-specific training in multiple rehabilitation services (e.g., occupational and physical therapy) may benefit from coordination of scheduling and therapy content. For example, occupational and physical therapies could be scheduled on alternating days, or with several hours of time between the two therapy sessions on a single day.

We propose that future research investigate consolidation and reconsolidation between rehabilitation sessions. Given the overwhelming evidence for the process of memory reconsolidation in declarative and procedural memories, it might be expected that individuals undergoing rehabilitation would also experience reconsolidation between sessions of therapy. For example, regaining independence in performing activities of daily living involves learning processes (Bayona et al., 2005). Importantly, older adults have been shown to benefit from reconsolidation (Corbin, 2017; Tassone et al., 2020) despite the known declines in consolidation related to healthy aging (Brown et al., 2009; Wilson et al., 2012; Fogel et al., 2014; Roig et al., 2014). However, one study found that reconsolidation was impaired in older adults with stroke relative to age-matched subjects without stroke (Censor et al., 2016), while other research has found that individuals with stroke, but not age-matched healthy controls, benefit from sleep-based consolidation of a motor task (Siengsukon and Boyd, 2008, 2009). Thus, further investigation into consolidation and reconsolidation among patient populations is warranted.

Recipients of rehabilitation would also benefit from the continued development of clinical protocols using non-invasive brain stimulation as an adjunct to enhance therapy-related memory consolidation and reconsolidation. Indeed, several studies regarding stroke rehabilitation have found benefits of pairing non-invasive brain stimulation with participation in rehabilitation (Khedr et al., 2005; Chang et al., 2010; Ilić et al., 2016; Rocha et al., 2016). In addition, transcranial direct current stimulation during wake (Reis et al., 2009, 2015; Sandrini et al., 2014) and during post-encoding sleep (Marshall et al., 2004, 2006; Göder et al., 2013; Westerberg et al., 2015), as well as repetitive transcranial magnetic stimulation during wake (Turriziani, 2012; Sandrini et al., 2013), have previously been shown to enhance memory consolidation and reconsolidation. Other sensory stimulation techniques such as targeted memory reactivation (Rasch et al., 2007; Oudiette and Paller, 2013; Shimizu et al., 2018; Johnson et al., 2019a, 2020; Hu et al., 2020) and rhythmic auditory stimulation (Ngo et al., 2013; Ong et al., 2016) have been used during post-encoding sleep to enhance consolidation.

In addition to task-specific memory modulation, future research should also focus on how to best induce generalization of skill between therapies in relation to the degree of memory stability and task similarity. For example, Mosha and Robertson (2016) had participants learn a word list and a motor skill, with overlapping rules to task elements, in quick succession and showed that generalization could be induced between the tasks (regardless of learning order) when the first memory was unstable. However, generalization did not occur when the memory for the first task was stabilized through the inclusion of a 2-h consolidation period. That is, generalization can occur to a Task B during instability of Task A, but such generalization can also come at the cost of retroactive interference to Task A (Robertson, 2018; Mutanen et al., 2020).

## Conclusions

Rehabilitation often involves learning. We first describe why clinicians should consider memory consolidation and reconsolidation. Secondly, we encourage future research to investigate how consolidation and reconsolidation relate to rehabilitation and translate previous work to decrease interference effects and enhance memory consolidation between rehabilitation sessions. Doing so may aid in the development of efficient and long-lasting interventions that are generalizable to clinically meaningful activities.

## Author Contributions

BJ: conceptualization, literature review, and manuscript writing. LC and KW: conceptualization and manuscript writing. All authors contributed to the article and approved the submitted version.

## Conflict of Interest

The authors declare that the research was conducted in the absence of any commercial or financial relationships that could be construed as a potential conflict of interest.
